# Pt_3_(CoNi) Ternary Intermetallic Nanoparticles Immobilized on N-Doped Carbon Derived from Zeolitic Imidazolate Frameworks for Oxygen Reduction

**DOI:** 10.3390/ma17194775

**Published:** 2024-09-28

**Authors:** Shiqi Song, Junhua Hu, Chupeng Wang, Mingsheng Luo, Xiaoxia Wang, Fengxia Zhai, Jianyong Zheng

**Affiliations:** 1 School of Materials Science and Engineering, East China University of Science and Technology, Shanghai 200237, China; 2 School of Mechanical and Power Engineering, East China University of Science and Technology, Shanghai 200237, China; 3 Sushui Energy Technology (Shanghai) Co., Ltd., Shanghai 200444, China; 4 Institute of Artificial Intelligence, Shanghai University, Shanghai 200444, China; zhengjy@shu.edu.cn

**Keywords:** Pt_3_(CoNi) intermetallic, proton exchange membrane fuel cell, oxygen reduction reaction, electrocatalysis, zeolitic imidazolate frameworks

## Abstract

Pt-based intermetallic compound (IMC) nanoparticles have been considered the most promising catalysts for oxygen reduction reaction (ORR) in proton exchange membrane fuel cells (PEMFC). Herein, we propose a strategy for producing ordered Pt_3_(CoNi) ternary IMC nanoparticles supported on N-doped carbon materials. Particularly, the Co and Ni are originally embedded into ZIF-derived carbon, which diffuse into Pt nanocrystals to form Pt_3_(CoNi) nanoparticles. Moreover, a thin layer of carbon develops outside of Pt_3_(CoNi) nanoparticles during the cooling process, which contributes to stabilizing the Pt_3_(CoNi) on carbon supports. The optimal Pt_3_(CoNi) nanoparticle catalyst has achieved significantly enhanced activity and stability, exhibiting a half-wave potential of 0.885 V vs reversible hydrogen electrode (RHE) and losing only 16 mV after 10,000 potential cycles between 0.6 and 1.0 V. Unlike the direct-use commercial carbon (VXC-72) for depositing Pt, we utilized ZIF-derived carbon containing dispersed Co and Ni nanocluster or nanoparticles to prepare ordered Pt_3_(CoNi) intermetallic catalysts.

## 1. Introduction

Proton-exchange membrane fuel cells (PEMFCs) have been considered the most promising energy conversion technology due to their advantages such as high energy efficiency, high energy density, and being environmentally friendly. Unfortunately, the sluggish kinetics of oxygen reduction reactions (ORRs) in the cathode need precious metal Pt as catalysts [1,2,3,4]. While the high cost and the performance degradation of Pt catalysts have hindered the wide-spread application of PEMFCs technology [5,6]. Consequently, it is of great importance to design Pt-based catalysts with high electrocatalytic activity and improved stability for ORR [7,8,9].

Intermetallic compounds (IMCs) are defined as a solid-state compound with ordered atomic structure and precise stoichiometry [10,11,12]. One of the most significant advantages for Pt-based IMCs is their effective bonding force to stabilize transition metals (M) through strong d–d orbital interactions with ordered Pt and M atoms [13,14]. This stabilization mechanism plays a crucial role in preventing the dissolution of M atoms into the electrolyte, and ensures the structural consistency and durability of catalytic activity centers [15,16]. Several kinds of Pt-based binary intermetallic catalysts, such as Pt_3_Co [17,18,19,20,21], PtNi [22], PtFe_3_ [23,24], etc., have been developed with excellent ORR catalytic performance. Currently, it is found that the incorporation of a third non-precious metal (M’) into bi-metal PtM intermetallic can further improve the synergistic effect between the components, enhancing catalytic performance and extending the life of catalysts [25,26,27]. Liang et al. [28] synthesized ordered L_10_-W-PtCo intermetallic nanoparticles, in which tungsten (W) served to stabilize the PtCo intermetallic structure, adapted the Pt-Pt distance, and optimized the binding energy between Pt and surface oxygen intermediates, resulting in enhanced ORR catalytic activity compared with random PtCo and regular L_10_-PtCo. The performance enhancement of Pt-M-M’ ternary IMCs offers promising prospects for advancing Pt-based ORR catalysts [29,30].

The catalyst supports are also crucial in promoting the activity and stability of catalysts [31,32]. For conventional Pt-based IMCs, the IMCs nanoparticles were deposited on the carbon supports by chemical or physical adsorption [33]. Weak bonding forces with carbon black supports often lead to the migration and degeneration of loaded nanoparticles [34]. It has been reported that improving the interaction between IMC nanoparticles and carbon supports is very significant for the improvement of catalysts’ stability [35]. The porous carbon supports with nitrogen doping, which is often synthesized by the thermal treatment of zeolitic imidazolate framework (ZIFs) crystals [36,37,38,39], could enhance the binding of Pt-based IMCs to the supports and effectively prevent the agglomeration of metal particles, thus optimizing the electrocatalytic performance [40,41,42]. Guo et al. [43] studied PtCo nanoparticles supported on Co-N-C, and the PtCo/Co-N-C showed exceptional performance, which took advantage of the enhanced metal–support interactions and alloying effects.

**Figure 1:**
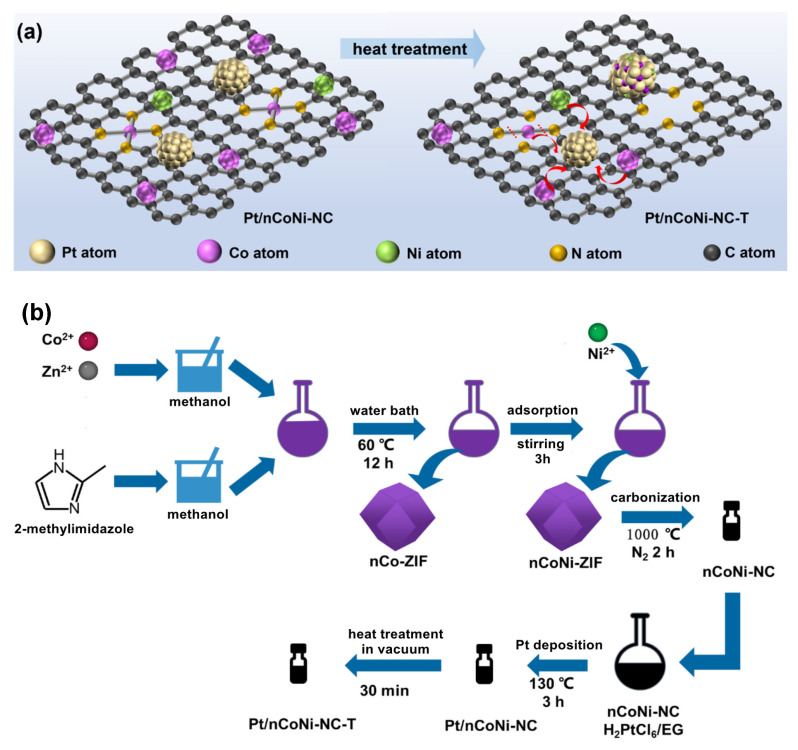
(**a**) Schematic illustration of the transformation process for the intermetallic Pt_3_(CoNi). (**b**) Schematic diagram of the synthesis for the Pt/nCoNi-NC-T.

Based on these significant studies, the synthesis of ternary intermetallic (Pt_3_(CoNi)) catalysts supported on nitrogen-doped carbon derived from ZIFs is proposed in this paper (Figure 1a). Herein, the atomic ratios of Co in the ZIFs’ precursor were adjusted to obtain a cobalt nanocluster/nanoparticle co-doped carbon skeleton structure and nickel was introduced by chemical adsorption. Pt nanoparticles were deposited on ZIF-derived carbon by ethylene glycol reduction. During the subsequent heat treatment, platinum was combined with cobalt and nickel to form Pt_3_(CoNi) IMC particles with a thin layer of carbon coating, which strengthened the interaction between the metal particles and carbon supports. Meanwhile, the nitrogen-doped carbon supports effectively inhibited the aggregation of nanoparticles, restricting the size of the Pt_3_(CoNi) particle. The optimized Pt/30CoNi-NC-700 demonstrate an excellent performance in terms of ORR catalytic activity and durability, attributed to the synergistic interaction between the IMCs and the carbon supports.

## 2. Materials and Methods

### 2.1. Synthesis of Co-ZIFs Precursors and CoNi-NC Supports

The co-doped ZIF precursors were prepared by a solution-phase method with different co-doping contents, designated as nCo-ZIF, where n% was defined as the molar percentage of Co of all metals (Co and Zn) in the starting materials. Typically, 4.76 g of Zn(NO_3_)_2_·6H_2_O (99%, Alfa Aesar, Shanghai, China) and 1.995 g of Co(NO_3_)_2_·6H_2_O (99%, Macklin, Shanghai, China) were dissolved in 400 mL of methanol (≥99.9%, Sinopharm Chemical Reagent Co., Ltd, Shanghai, China). Then, 7.88 g of 2-methylimidazole (≥99%, Macklin) was dissolved in another 400 mL of methanol. Next, the two well-stirred solutions were quickly mixed together, heated in water bath at 60 °C for 12 h. The resulting purple deposits were collected by centrifugation, washed with ethanol (95%, Sinopharm Chemical Reagent Co., Ltd) three times, and dried in a vacuum oven (DZF, Shanghai Kuntian Laboratory Instrument Co., Ltd., Shanghai, China) at 60 °C for 10 h; the 30Co-ZIF was acquired. The CoNi-ZIF precursors were obtained via an adsorption method, as reported by the previous publication, in which Co-ZIF was used as support and Ni^2+^ were inserted to generate CoNi-ZIF precursors [44,45,46]. Typically, 2.5 g of the Co-ZIF precursor was dispersed in 250 mL of methanol at first. Then, 1.25 mL methanol solution of Ni(NO_3_)_2_·6H_2_O (98%, Shanghai Lingfeng Chemical Reagent Co., Ltd, Shanghai, China) (100 mg/mL) was dropped carefully into the Co-ZIF suspension and the mixed solution was stirred for 3 h. The precipitates were then collected by centrifugation, washed with ethanol three times, and dried in a vacuum oven at 65 °C for 8 h. The Co and Ni co-doped CoNi-ZIF precursors were obtained. Next, the CoNi-ZIF powder were pyrolyzed in a porcelain boat within a tube furnace (OTF-1200X, Hefei Kejing Materials Technology Co., Ltd, Hefei, China) under an N_2_ atmosphere at 1000 °C for 2 h at a ramp rate of 5 °C min^−1^. Carbon supports doped with Co, Ni, and N were obtained and labeled as nCoNi-NC.

### 2.2. Synthesis of Pt/nCoNi-NC and Ternary IMCs Catalysts

The Pt/nCoNi-NC catalysts were synthesized by an ethylene glycol reduction method. Typically, 120 mg of nCoNi-NC powder was dispersed in 240 mL ethylene glycol (99.7%, Sinopharm Chemical Reagent Co., Ltd) by ultrasonic dispersion. Then, 21.2 mL of 10 mg_Pt_/mL H_2_PtCl_6_·6H_2_O (98%, Sinopharm Chemical Reagent Co., Ltd) in EG was added into the mixture, heated, and refluxed in an oil bath at 130 °C for 3 h. The resulting products were then collected by filtration, washed with ultra-pure water three times, and dried in a vacuum oven at 80 °C for 8 h. The Pt/nCoNi-NC catalysts with 40 wt% Pt loading were obtained. The Pt/30CoNi-NC was chosen to prepare IMCs catalysts. Typically, 50 mg of Pt/30CoNi-NC was sealed in a small vacuum quartz tube, which was put into another quartz tube and subjected to annealing treatments at 600 °C, 700 ° C, 800 °C, and 900 °C for 0.5 h (5 °C min^−1^) under a flowing N_2_ atmosphere to obtain Pt/30CoNi-NC-T (T = 600, 700, 800, 900), respectively. The preparation process of ternary IMCs Pt/nCoNi-NC-T was presented in Figure 1b.

### 2.3. Material Characterization

X-ray diffraction patterns of all samples were collected using an X-ray diffractometer with Cu-Kα radiation (XRD, Rigaku Corporation (Tokyo, Japan), D/max 2550 VB/PC, λ = 0.154056 nm). The morphologies and structures of the samples were observed on a transmission electron microscope (TEM) and high-resolution TEM (HRTEM, FEI Company (Hillsboro, OR, USA), Tecnai F20) equipped with energy-dispersive spectroscopy (EDS). Surface elemental composition and valence state of the samples were carried out using an X-ray photoelectron spectroscope (XPS, Thermo Scientific (Waltham, MA, USA), Escalab 250Xi). The weight percentage of elements in the samples was determined by an inductively coupled plasma optical emission spectrometer (ICP-OES, Agilent Technologies Inc. (Santa Clara, CA, USA), 725).

### 2.4. Electrocatalytic Measurements

The electrocatalytic performance of the samples was evaluated on a standard three-electrode configuration by a CHI760E electrochemical workstation. A Hg/Hg_2_SO_4_ and a platinum wire were used as the reference electrode and the counter electrode, respectively. The rotating ring-disk electrode (RRDE) consisted of a glassy carbon (GC) disk (geometric surface area of 0.2463 cm^2^) surrounded by a Pt ring. A GC electrode modified with the studied catalysts was used as the working electrode. The electrolyte was 0.1 M HClO_4_ solution prepared by deionized water. The catalyst ink was obtained by ultrasonically dispersing 5 mg of the catalyst into the mixed solution of 980 μL isopropyl alcohol and 20 μL Nafion (5 wt%). Then, 7.5 μL ink was drawn by a pipette and dropped onto the surface of GC electrode and dried naturally to form a uniform thin film. The mass loading of Pt on the GC electrode was 60 μg_Pt_ cm^−2^.

The recorded potentials were calibrated to the reversible hydrogen electrode (RHE) using the equation: E (RHE) = E (Hg/Hg_2_SO_4_) + 0.059 × pH + 0.658 (V). The samples were first activated by cyclic voltammetry (CV) at a scan rate of 50 mV s^−1^ in N_2_-saturated 0.1 M HClO_4_ for 50 cycles. Then, linear scanning voltammetry (LSV) testing was performed at a scan rate of 5 mV s^−1^ in O_2_-saturated 0.1 M HClO_4_ with the electrode rotated from 400 to 2500 rpm. The electron transfer number (n) in the ORR was calculated by the Koutecky–Levich equation:

(1) $$\frac{1}{j}=\frac{1}{j_{k}}+\frac{1}{j_{d}}=\frac{1}{j_{k}}+\frac{1}{0.62nFC_{O}{D_{O}}^{2/3}\nu^{-1/6}\omega^{1/2}}$$

where *j*, *j_k_*, and *j_d_* were the measured current density, kinetic, and diffusion limiting current densities (mA cm^−2^), respectively; *F*: the Faraday constant (96,485 C mol^−1^), *D_O_*: the diffusion coefficient of O_2_ in 0.1 M HClO_4_ (*D_O_* = 1.93 × 10^−5^ cm^2^ s^−1^), *C_O_*: the concentration of O_2_ dissolved in 0.1 M HClO_4_ (*C_O_* = 1.26 × 10^−3^ mol L^−1^), *ν*: the kinetic viscosity of 0.1 M HClO_4_ (*ν* = 0.01 cm^2^ s^−1^), and *ω* the electrode rotation speed (rad s^−1^). The accelerated durability tests (ADTs) were performed in O_2_-saturated 0.1 M HClO_4_ solutions by using the cyclic potential sweeps between 0.6 and 1.0 V vs. RHE at a scan rate of 50 mV s^−1^ for 10,000 cycles, and the CV and LSV curves were recorded after 2000, 4000, 8000, and 10,000 cycles, respectively. Commercial J01-Pt/C (HiSPEC4000, Johnson Matthey (London, UK)) catalyst with 40 wt% Pt content was used as the comparison test, and the working electrode preparation process and test conditions were same as above.

To measure the electrochemically active surface area (ECSA) of the catalyst, the stable CV curve after activation was selected and its hydrogen desorption peak was integrated to obtain *S_H_*, then the ECSA was derived from the following equation:

(2) $$ECSA=\frac{S_{H}/V}{Q_{AC}\cdot m_{\mathrm{Pt}}}$$

where *S_H_* represented the total charge integral area corresponding to the hydrogen desorption peak after the removal of the double layer region, *Q_AC_* was the theoretical reference value for the adsorption of hydrogen in a single layer of Pt in acid (*Q_AC_* = 0.21 mC cm^−2^), *V* indicated the scanning speed (mV s^−1^), and *m*_Pt_ expressed the mass of Pt on the electrode (g). Specific activity (*S_A_*) was also the primary parameter for measuring the catalytic activity of the catalysts, which was calculated using the following formula:

(3) $$S_{A}=\frac{j_{k}}{m_{\mathrm{Pt}}\cdot ECSA}$$

## 3. Results

### 3.1. Phase Transformation from Pt to Pt_3_(CoNi) during the Preparation Process

**Figure 2:**
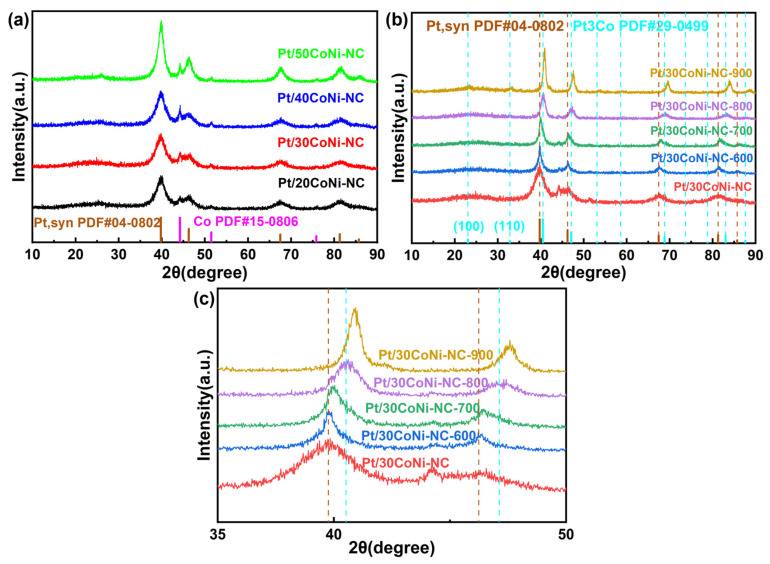
XRD patterns of the (**a**) Pt/nCoNi-NC, (**b**) Pt/30CoNi-NC-T catalysts, (**c**) enlarged region of 2θ from 35° to 50° for Pt/30CoNi-NC-T catalysts. The brown dashed line and blue dashed line correspond to the peak position of pure Pt and Pt_3_Co intermetallic, respectively.

The XRD patterns for nCoNi-NC carbon supports are shown in Figure S1. The broad peaks at 25° for all samples corresponded to the (002) diffraction of graphite, the peaks at 2θ = 44.22°, 51.52°, and 75.85° correspond to the (111), (200), and (220) diffractions of the metal Co, respectively. With the increase in Co content, the peaks became stronger and sharper, indicating larger particle sizes and the greater crystallization of Co nanoparticles. No obvious Ni metal peak was observed, which can be explained by the low Ni content. The XRD results showed that nCoNi-ZIF precursors had transformed into carbon materials with Co nanoparticles buried in a carbon matrix after carbonization. After Pt deposition, the diffraction peaks for Pt could be obviously found, indicating that Pt nanoparticles were successfully loaded onto carbon carriers (Figure 2a). The diffraction peaks at 2θ = 39.76°, 46.24°, and 67.45° were consistent with the pure Pt metal with an fcc structure, corresponding to the (111), (200), and (220) planes. It should be noted that the diffraction peaks for Pt or Co did not shift, which demonstrated that the Co nanoparticles and Pt nanoparticles were independent units without forming alloy phase. The ICP analysis results (Table S1) further confirmed the co-existence of Co, Ni, and Pt in Pt/30CoNi-NC, and the Pt content (36.191 wt%) was close to the theoretical value (40 wt%), indicating that most Pt elements in the precursor are well reduced onto the carbon carriers.

Figure 2b illustrated the X RD patterns of the Pt/30CoNi-NC catalysts after heat treatment at different temperatures. As shown, the diffraction peaks at 2θ = 39.76° and 46.24° for Pt began to shift to higher angels slightly. With the increase in heat temperature, the positive shift of these two peaks became more and more obvious and the peaks for Co disappeared gradually, indicating a phase transformation from Pt to Pt_3_Co [47,48]. The diffraction peaks of Pt/30CoNi-NC-700 shifted to a higher angle between the peaks of Pt and Pt_3_Co, which indicated a transformation of the alloy from a disordered to an ordered phase, forming a partially ordered Pt_3_(CoNi). At a heat temperature of 800 °C, the diffraction peaks for Pt/30CoNi-NC-800 were completely consistent with those of standard Pt_3_Co, which developed a fully ordered structure (Figure 2c) [18]. The superlattice diffraction peaks associated with the (100) and (110) crystal planes of the Pt_3_Co cannot be clearly identified due to the influence of the (002) broad peak of graphite. While if the heat temperature increased higher than 800 °C, the peak at 2θ = 46.24° shifted positively continuously, indicating more Co and Ni atoms substituted or doped in Pt with lattice contraction.

**Figure 3:**
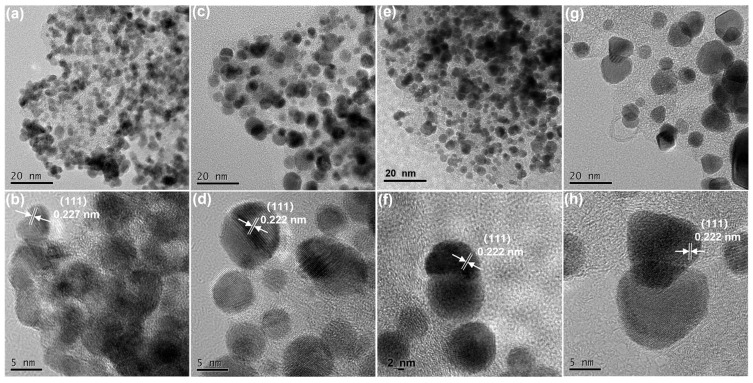
TEM and HRTEM images of different catalysts. (**a**,**b**) Pt/30CoNi-NC, (**c**,**d**) Pt/30CoNi-NC-600, (**e**,**f**) Pt/30CoNi-NC-700, and (**g**,**h**) Pt/30CoNi-NC-800.

TEM and HRTEM were employed to further characterize the morphology of Pt/30CoNi-NC and Pt/30CoNi-NC-T (Figure 3). The Pt nanoparticles dispersed on the Co and Ni co-doping ZIF-derived carbon supports uniformly, as shown in Figure 3a, with an average particles size of about 2.7 nm (Figure S2a). The particle size increased with the increasing temperature (Figure 3c,e,g), resulting from the sintering and aggregation of the particles. The average particle size for the catalysts heated at 600, 700, and 800 °C were 5.24, 6.53, and 8.83 nm, respectively (Figure S2b–d). Unfortunately, the increase in particle size would reduce the active surface of catalysts, ultimately reduce catalytic performance. The HRTEM images of Pt/30CoNi-NC and Pt/30CoNi-NC-600 shown in Figure 3b,d displayed lattice spacings of 0.227 and 0.222 nm, corresponding to the (111) plane of metal Pt and (111) plane of ordered Pt_3_Co; this result showed that the metal particles began to transition to the IMC at 600 °C, which agreed well with the XRD characterization results. The lattice spacing corresponding to the (111) plane of Pt_3_Co was also indicated in the HRTEM images of Pt/30CoNi-NC-700 and Pt/30CoNi-NC-800 (Figure 3f,h). Furthermore, the elemental scanning images of Pt/30CoNi-NC-700 (Figure S3) demonstrated the synchronous distribution of Pt, Co, Ni, and N on the carbon carriers, suggesting that Co and Ni atoms diffused into Pt nanoparticles during heat treatment.

### 3.2. Characterization of Microstructure of the Pt and Pt_3_(CoNi) Nanoparticles

The valence states of C, N, Pt, and Co in Pt/30CoNi-NC before and after heat treatment were characterized by XPS. The full spectrum (Figure S4) showed no obvious Ni 2p peak due to the low Ni doping amount. The surface elemental composition of the catalysts was included in Table S2. As shown, the Pt content decreased, while the Co content increased with the increase in heat temperature. This change initially proved that the Pt in Pt/30CoNi-NC was deposited on the surface of the carbon supports and most of the Co nanoparticles were covered. As the heat temperature increased, the Co nanoparticles in the carbon base diffused and migrated to combine with the Pt nanoparticles on the surface, resulting in the formation of the IMC Pt_3_(CoNi). The XPS data illustrated higher Ni content on the catalyst surface than the ICP results, suggesting that most Ni was in the outermost layer of the catalyst. The Pt/30CoNi-NC-700 and Pt/30CoNi-NC-800 showed an atomic ratio of Pt to (Co+Ni) close to 3:1, which confirmed the formation of Pt_3_(CoNi) again.

**Figure 4:**
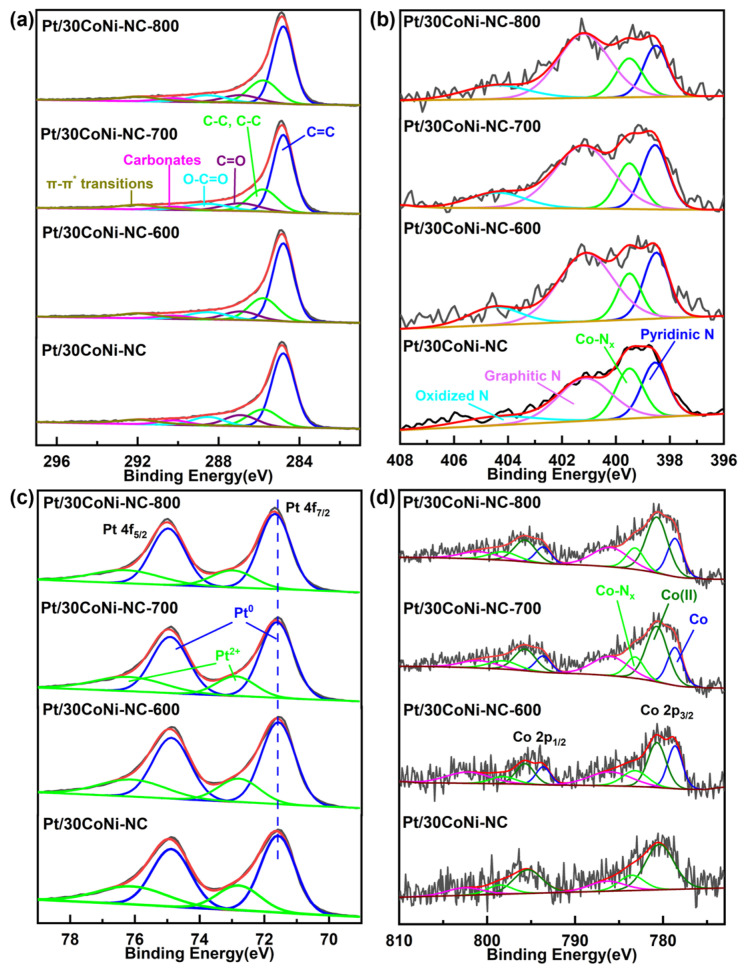
XPS high resolution spectrum of (**a**) C 1s, (**b**) N 1s, (**c**) Pt 4f, (**d**) Co 2p for Pt/30CoNi-NC and Pt/30CoNi-CN-T.

In Figure 4a, the C 1s spectrum could be deconvoluted into C–C/C=C, C–O and C=O peaks. After heat treatment at different temperatures, the spectrum for carbon stayed almost unchanged, indicating its stable structure [49]. It should be noted that the content of C increased from 66.98% for the sample before heat treatment to 79.56% for Pt/30CoNi-NC-800. And the content of Pt decreased from 16.22% for the Pt/30CoNi-NC to 5.83% for Pt/30CoNi-700, while that for Co increased from 1.03% to 2.11%, correspondingly (Table S2). Before heat treatment, the surface of the carbon supports was deposited with Pt nanoparticles. During heat treatment, Co nanoparticles in the carbon supports diffused, migrated, and combined with Pt nanoparticle, with the formation of a Pt_3_Co intermetallic compound, as discussed in the XRD results. In this process, more Co and C diffused on the surface of catalysts.

For the catalysts before and after heat treatment, the N 1s spectra (Figure 4b) were deconvoluted into four peaks at 398.6 eV (pyridinic N), 399.5 eV (Co-N_x_), 401.1 eV (graphitic N), and 404.1 eV (oxidized N). It could be found that the content of N decreased with the increase in heat temperature (Table S2), indicating the breaking of part Co-N_x_ bonds and the removal of N atoms. At the same time, the percentage content of Co-N_x_ decreased after heat treatment, further proving the crack of Co-N_x_ bonds at high temperature (Table S3) [21,43].

Pt 4f spectra were fitted into two groups of peaks representing Pt^0^ and Pt^2+^ (Figure 4c). Table S4 showed that the integration area of the characteristic peak of Pt^0^ was larger than that of Pt^2+^, which indicated that the Pt on the surface of the catalyst was mainly present in the metallic state. With the increase in heat temperature, the metal Pt^0^ content increased while that of Pt^2+^ decreased, which might be due to the changes in electronic structure, indicating that alloying Pt with Co and Ni could reduce the oxophilicity of Pt [50]. The larger proportion of Pt^0^ species could provide more free Pt sites for O_2_ adsorption/desorption, contributing to increased ORR activity [51]. It was acknowledged that the binding energy (BE) was correlated with the adsorption/desorption behavior of oxygen species on the catalyst surface [52], and the interaction between oxygen species and Pt was affected by the d-band center. The BE of Pt^0^ 4f_7/2_ in Pt/30CoNi-NC-600 (71.57 eV) was very close to that in Pt/30CoNi-NC (71.58 eV), while the Pt^0^ 4f_7/2_ peak of the Pt/30CoNi-NC-700/800 shifted to higher BEs (71.61/71.67 eV). The change in BE could be ascribed to electronic interactions between Pt, Co, and Ni atoms with diverse electronegativities, and compressive strains caused by Co and Ni doping, decreasing the interatomic distance between Pt atoms and inducing a widened d-band width. The position of the d-band center with respect to the Fermi energy level was lowered, and thus the adsorption energy was reduced [53,54,55]. The compression of the lattice spacing in Pt/30CoNi-NC-700 led to a negative shift of the d-band center compared with that of Pt/30CoNi-NC, and the adsorption between Pt and oxygen species was weakened, which balanced the adsorption/desorption behaviors of oxygen species on the surface of catalysts and released more active sites to accelerate ORR kinetics [56,57,58]. This suggested that the electronic structure of Pt on the surface of Pt/30CoNi-NC-700 was more favorable for ORR, and thus partially ordered Pt_3_CoNi exhibited excellent catalytic performance. The Co 2p spectrum (Figure 4d) showed a characteristic peak of metallic valence Co in Pt/30CoNi-NC-T, which suggested that Co species have migrated into the Pt nanolattice during heat treatment to form lattice-shrinking IMCs [59,60]. In addition, the nitrogen atoms in the carbon supports were also coordinated with Co species during the heat treatment [61]. Consequently, both the compressive strain and electronic structure effects would contribute to the increased intrinsic ORR catalytic activity.

### 3.3. Electrochemical Measurement Results

The Pt/nCoNi-NC catalysts’ electrocatalytic activities were tested first. As shown in Figure S5a, Pt/30CoNi-NC exhibited the best ORR performance in 0.1 M HClO_4_ with the onset potential (E_onset_) of 0.961 V and half wave potential (E_1/2_) of 0.851 V. The reason for this might be the appropriate Co content in the ZIF-derived carbon supports. When the content of Co exceeded 30%, the ORR catalytic activity decreased due to the large amount and agglomeration of Co nanoparticles. The ORR catalytic activity of Pt/30Co-NC was also tested, as shown in Figure S5b. The half-wave potential for Pt/30Co-NC was 0.846 V vs. RHE, a little lower than that of Pt/30CoNi-NC (0.851 V vs. RHE). Based on these results, the Pt/30CoNi-NC catalyst was then submitted to heat treatment in order to study the transformation process of the ordered structure and the ORR catalytic performance.

**Figure 5:**
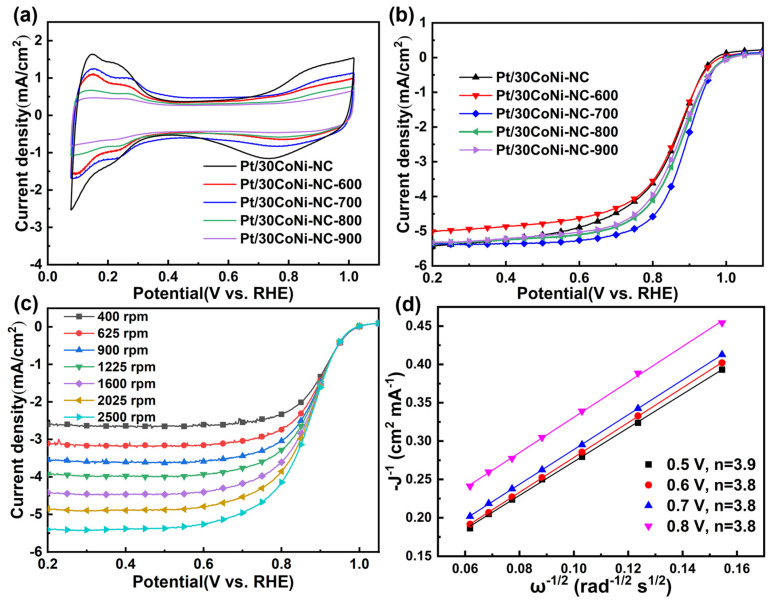
(**a**) CV curves and (**b**) ORR polarization curves of Pt/30CoNi-NC-T, (**c**) LSV curves of Pt/30CoNi-NC-700 with different rotational speeds, and (**d**) corresponding Koutecky–Levich plots with linear fitting.

The ORR performance of the Pt/30CoNi-NC and Pt/30CoNi-NC-T were investigated by a typical three-electrode system in O_2_-saturated 0.10 M HClO_4_. Figure 5a showed the cyclic voltammetry (CV) curves of these catalysts, and the electrochemical active area (ECSA) could be calculated by the desorption peak area of hydrogen (deducting the charge contribution from the electric double layer), and the results are listed in Table S5. For the samples before and after heat treatment, Pt/30CoNi-NC had the largest ECSA because of its smaller Pt particle size than those of Pt/30CoNi-NC-T. The LSV curves of the catalysts are shown in Figure 5b. It could be seen that the activity for ORR was similar for Pt/30CoNi-NC and Pt/30CoNi-NC-600, and the partially ordered Pt/30CoNi-NC-700 exhibited the best catalytic activity with an E_1/2_ of 0.885 V vs. RHE. While the catalytic activities for the catalysts heated at 800 and 900 °C reduced, they were still higher than those before heat treatment. According to the XRD results, the catalysts annealing at 800 °C showed a fully ordered structure of Pt_3_(CoNi), but its performance was lower than that heated at 700 °C. The main reason for this might be the bigger particle size during the higher heat temperature. Therefore, both the ordering degree of the alloy and the size of the nanoparticles must be considered in order to obtain the best performance. Figure 5c displayed the ORR polarization curves of Pt/30CoNi-NC-700 at different rotation speeds. The corresponding Koutecky–Levich (K-L) plots for Pt/30CoNi-NC-700 (Figure 5d) showed good linearity at 0.5–0.8 V vs. RHE, and the electron transfer number was 3.8~3.9, which indicated that the catalytic reaction on the surface of Pt/30CoNi-NC-700 followed a 4-electron pathway with high energy conversion efficiency. Consequently, the heat temperature was critical for the formation of the IMCs, which had a significant effect on IMCs’ catalytic activity for ORR. The catalytic activity of the partially ordered Pt/30CoNi-NC-700 was attributed to the small nanoparticle size and the presence of IMCs. Co and Ni doping induced a dual optimization of the electronic and geometrical structure of the catalyst surface, generating a uniform distribution of active sites and simultaneously increasing the catalytic activity for ORR.

**Figure 6:**
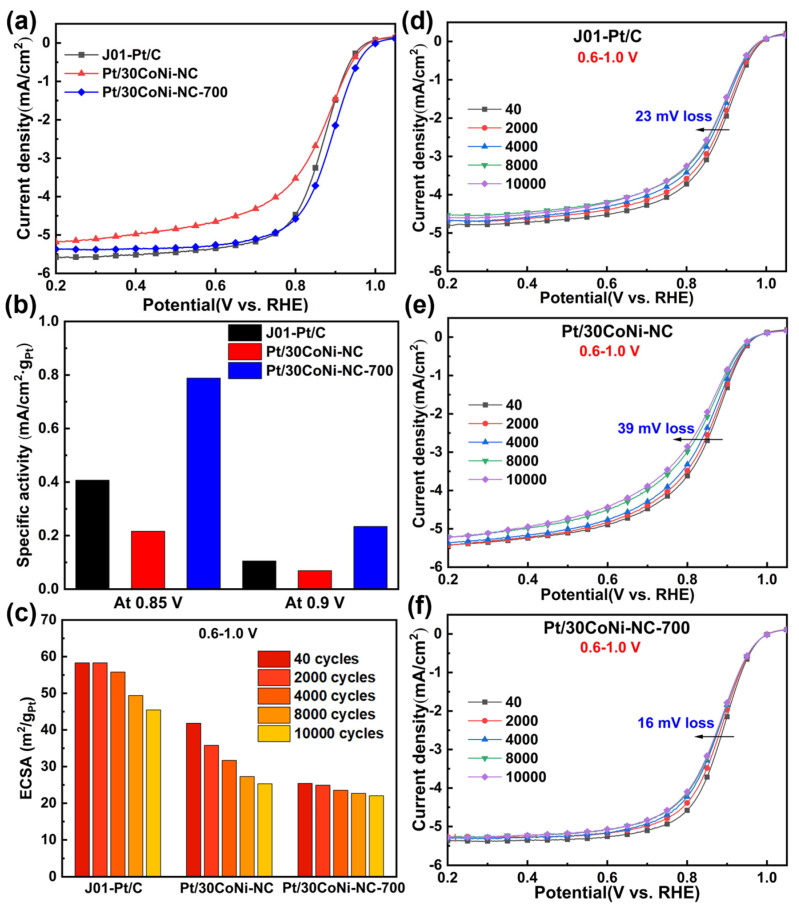
(**a**) ORR polarization curves, (**b**) specific activity comparison chart, and (**c**) ECSA retention of J01-Pt/C, Pt/30CoNi-NC, and Pt/30CoNi-NC-700 during the ADTs, (**d**–**f**) LSV curves at different interval cycles of ADTs test at 0.6–1.0 V.

Figure 6a showed that the E_1/2_ of J01-Pt/C, Pt/30CoNi-NC, and Pt/30CoNi-NC-700 were 0.863 V, 0.851 V, and 0.885 V, respectively. And the E_onset_ of Pt/30CoNi-NC-700 (0.988 V) was 23 mV higher than that of J01-Pt/C, indicating that it had a higher ORR catalytic activity than commercial Pt/C. Pt/30CoNi-NC-700 exhibited the highest area specific activities (SA) (Figure 6b). At 0.9 V, the SA of Pt/30CoNi-NC-700 was about 2 times higher than that of J01-Pt/C, indicating the high intrinsic catalytic activity of Pt_3_(CoNi).

For practical application in fuel cells, stability is significantly important. The stability of the three samples were measured by the accelerated degradation tests (ADTs) via scanning the potential from 0.6 to 1.0 V (vs. RHE). The initial ECSAs of J01-Pt/C and Pt/30CoNi-NC were larger than that of Pt/30CoNi-NC-700 (Figure S6). During the ADTs, the CV curves at different intervals were recorded and shown in Figure S7. The ECSAs decreased with the increase in cycling number. After 10,000 cycles, the ECSAs of J01-Pt/C, Pt/30CoNi-NC, and Pt/30CoNi-NC-700 decreased by 22.0%, 39.5%, and 13.3% (Figure 6c), respectively. Figure 6d–f showed that the E_1/2_ of the three catalysts dropped by 23 mV, 39 mV, and 16 mV after 10,000 cycles, respectively, which demonstrated the improved stability of the Pt/30CoNi-NC-700 catalyst for ORR. The formation of partial IMCs of Pt with Co and Ni changed the electronic and geometric structures on the surface of the nanoparticles. Moreover, these nanoparticles with ordered structure were dispersed on the nitrogen-doped carbon supports, resulting in the best ORR catalytic performance of Pt/30CoNi-NC-700.

## 4. Conclusions

In conclusion, we have succeeded in synthesizing a high catalytic activity and stability catalyst, in which the Pt, Co, and Ni ternary IMC nanoparticles were anchored on the ZIF-derived nitrogen-doped carbon supports. The optimal partially ordered Pt_3_(CoNi) catalyst (Pt/30CoNi-NC-700) exhibited excellent ORR catalytic performance in acidic media due to the electronic and geometric effects arising from the intermetallic compound structure of Pt_3_(CoNi) and the synergistic effect of the fine nanoparticles and nitrogen-doped carbon supports. The E_1/2_ of Pt/30CoNi-NC-700 was 0.885 V vs. RHE, and dropped only 16 mV after 10,000 cycles of ADTs at 0.6–1.0 V. Our work provided an effective and convenient method for the fabrication of ternary-ordered Pt alloy composite catalysts with small particle size and carbon coating, which was also applicable to the synthesis of other Pt-based alloy catalysts.

## Data Availability

The original contributions presented in the study are included in the article/Supplementary Materials; further inquiries can be directed to the corresponding authors.

## Supplementary Materials

The following supporting information can be downloaded at: https://www.mdpi.com/article/10.3390/ma17194775/s1, Figure S1: XRD patterns of nCoNi-NC carbon supports; Figure S2: Particle size distribution histogram of (a) Pt/30CoNi-NC, (b) Pt/30CoNi-NC-600, (c) Pt/30CoNi-NC-700 and (d) Pt/30CoNi-NC-800; Figure S3: (a) HAADF-STEM image and (b–f) corresponding elemental mappings of Pt/30CoNi-NC-700; Figure S4: XPS wide scanning spectrum of Pt/30CoNi-NC before and after heat treatment at different temperatures; Figure S5: ORR polarization curves of (a) Pt/nCoNi-NC catalysts, (b) Pt/30CoNi-NC and Pt/30Co-NC; Figure S6: CV curves of J01-Pt/C, Pt/30CoNi-NC and Pt/30CoNi-NC-700 catalysts; Figure S7: Cyclic voltammetry curves of (a) J01-Pt/C, (b) Pt/30CoNi-NC and (c) Pt/30CoNi-NC-700 recorded during ADTs; Table S1: The ICP-OES results of the Pt/30CoNi-NC; Table S2: Elemental composition of different catalysts by XPS; Table S3: Summary of fitting results for N 1s XPS spectra for different catalysts; Table S4: Summary of fitting results for Pt 4f XPS spectra of different catalysts; Table S5: ECSA of the samples before and after heat treatment.
