# Supplementary material for: Pt3(CoNi) Ternary Intermetallic Nanoparticles Immobilized on N-Doped Carbon Derived from Zeolitic Imidazolate Frameworks for Oxygen Reduction
Source: Materials (Basel). 2024 Sep 28;17(19):4775. doi: 10.3390/ma17194775 (PMC11477947; doi:10.3390/ma17194775)
Supplement: Supplementary file 1 [file materials-17-04775-s001.zip › materials-3189103-supplementary.pdf]

Supporting Information

$Pt_3(CoNi)$ ternary intermetallic compounds nanoparticles immobilized on N-doped carbons derived from zeolitic imidazolate frameworks for oxygen reduction

Shiqi Song[1], Junhua Hu[1], Chupeng Wang[1], Mingsheng Luo[1], Xiao Xia Wang[1,*] Fengxia Zhai[2,*]

[1]School of Mechanical and Power Engineering, East China University of Science and Technology, Shanghai 200237, China

*Corresponding author

Xiao Xia Wang: wangxiaoxia@ecust.edu.cn

Fengxia Zhai: zfx@sushuiny.com

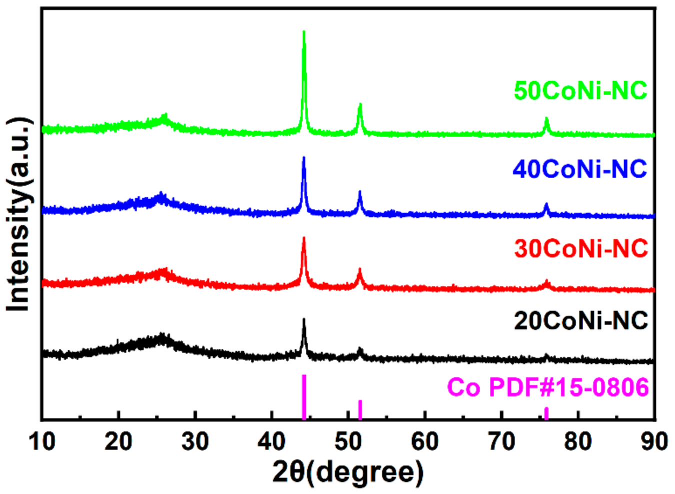

Figure S1. XRD patterns of nCoNi-NC carbon supports.

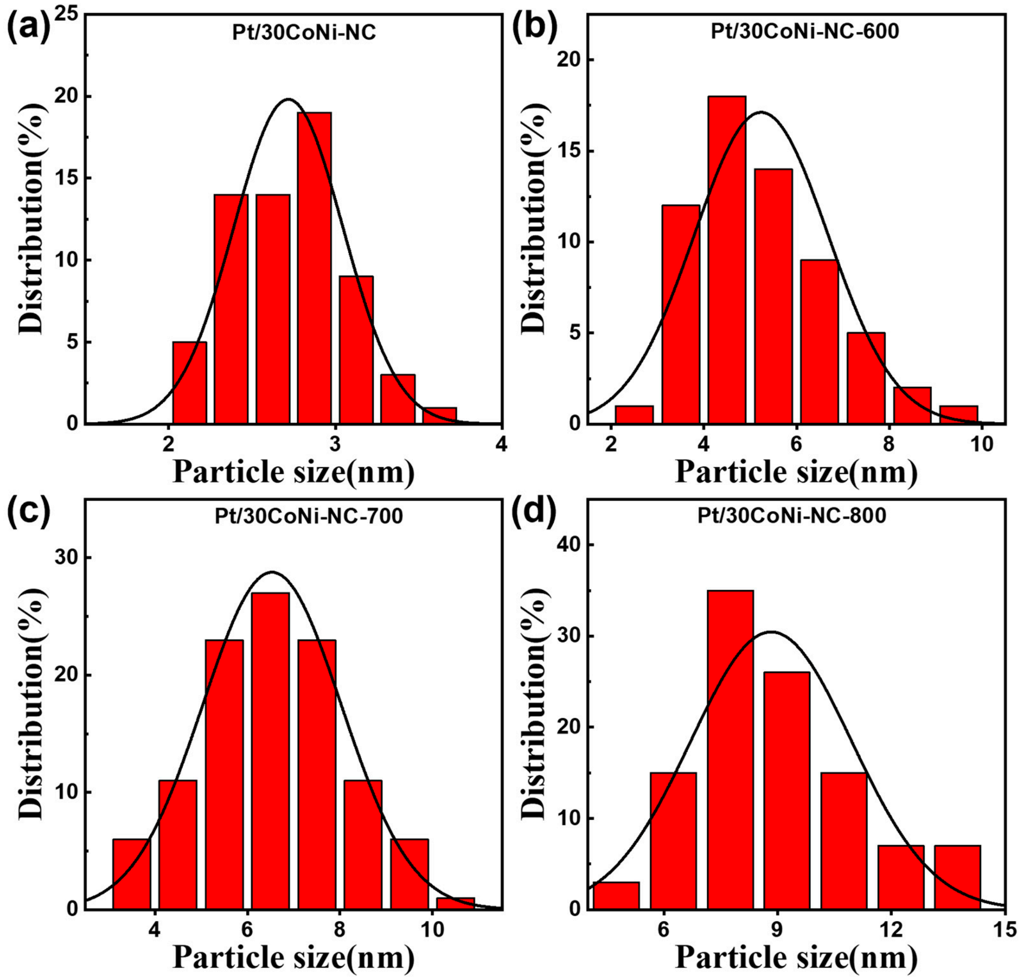

Figure S2. Particle size distribution histogram of (a) Pt/30CoNi-NC, (b) Pt/30CoNi-NC-600, (c) Pt/30CoNi-NC-700 and (d) Pt/30CoNi-NC-800.

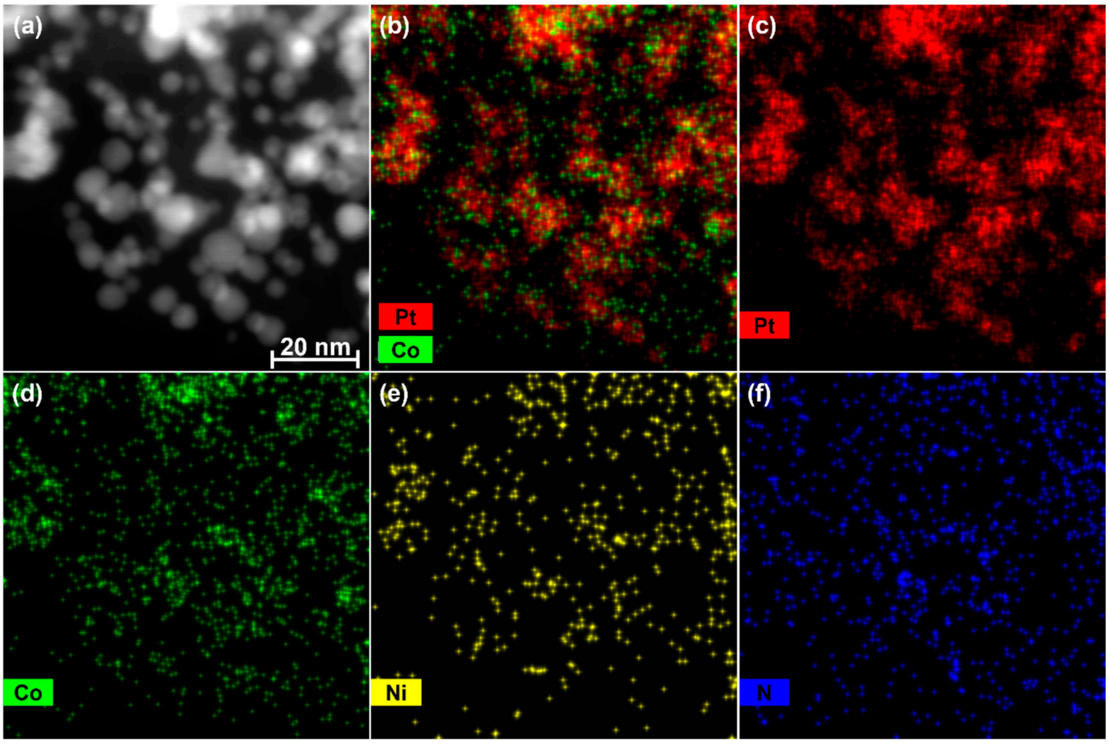

Figure S3. (a) HAADF-STEM image and (b-f) corresponding elemental mappings of Pt/30CoNi-NC-700.

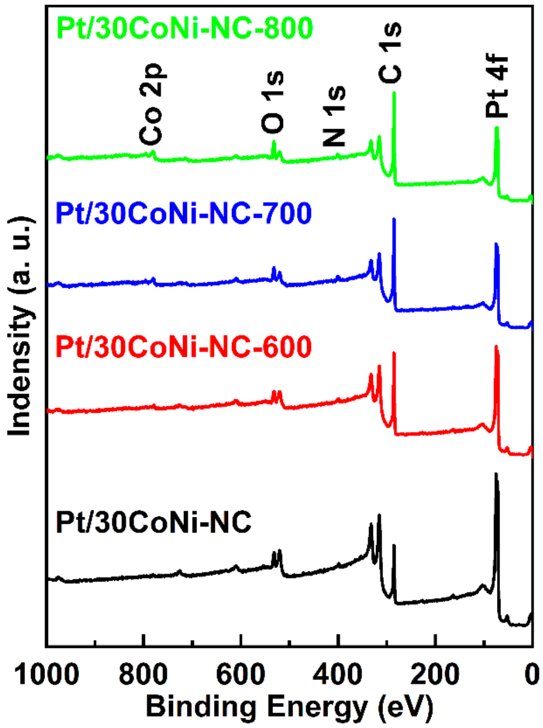

Figure S4. XPS wide scanning spectrum of Pt/30CoNi-NC before and after heat treatment at different temperatures.

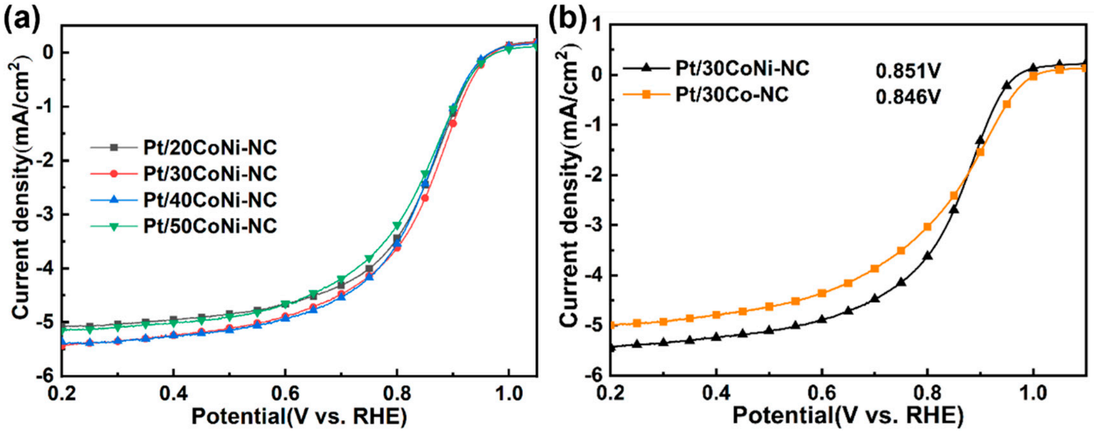

Figure S5. ORR polarization curves of (a) Pt/nCoNi-NC catalysts, (b) Pt/30CoNi-NC and Pt/30Co-NC.

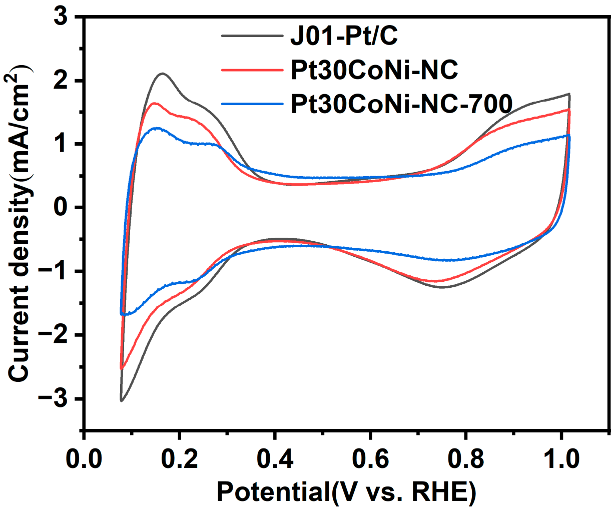

Figure S6. CV curves of J01-Pt/C, Pt/30CoNi-NC and Pt/30CoNi-NC-700 catalysts**.**

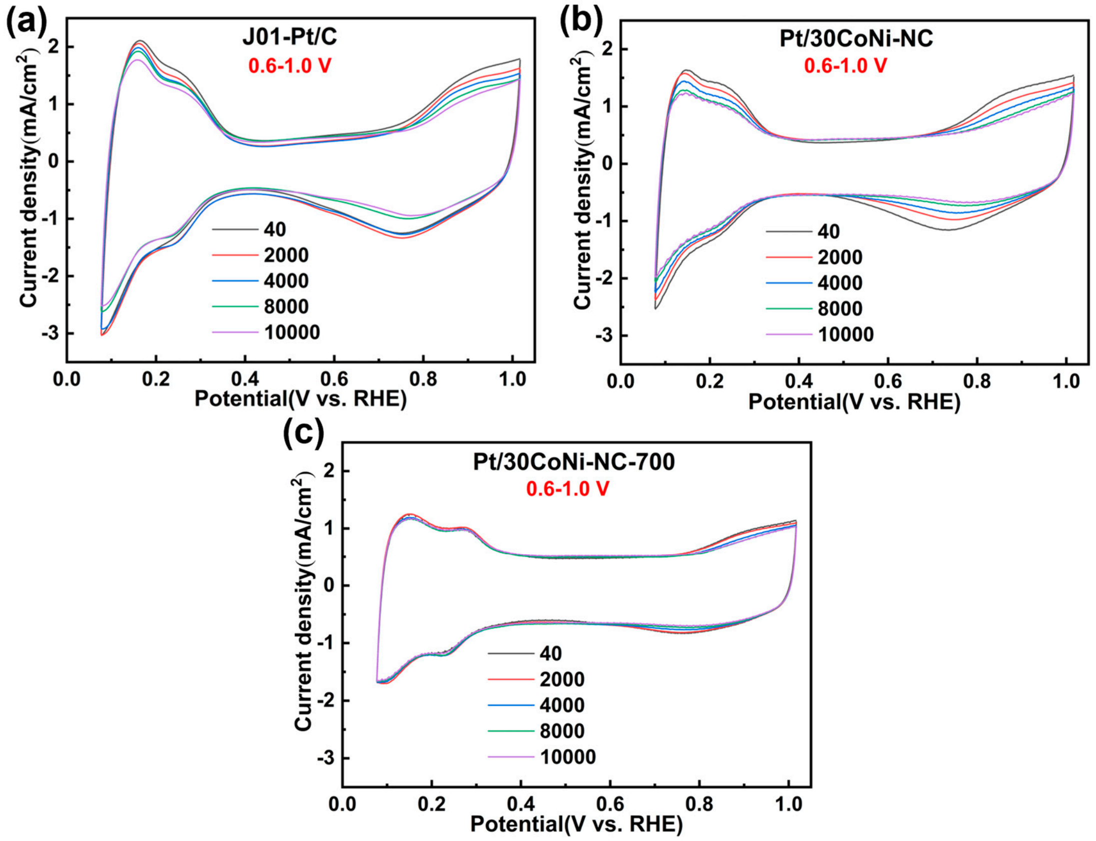

Figure S7. Cyclic voltammetry curves of (a) J01-Pt/C, (b) Pt/30CoNi-NC and (c) Pt/30CoNi-NC-700 recorded during ADTs.

Table S1. The ICP-OES results of the Pt/30CoNi-NC.

Sample	Element	The Ratio of Atomic Mole Feeding radio (result from ICP-OES) wt%
Pt/30CoNi-NC	Pt	36.191
	Co	1.895
	Ni	0.004

Table S2. Elemental composition of different catalysts by XPS.

Samples	C (at%)	N (at%)	O (at%)	Pt (at%)	Co(at%)	Ni (at%)
Pt/30CoNi-NC	66.98	5.49	9.7	16.22	1.03	0.59
Pt/30CoNi-NC-600	76.98	5.36	6.56	9.49	1.08	0.53
Pt/30CoNi-NC-700	79.18	4.73	7.39	6.52	1.6	0.58
Pt/30CoNi-NC-800	79.56	3.74	8.3	5.83	2.11	0.46

Table S3. Summary of fitting results for N 1s XPS spectra for different catalysts

Samples	Relative intensity (%)			
	pyridinic-N	$Co\text{-}N_x$	graphitic-N	oxidized N
Pt/30CoNi-NC	23.41	22.2	39.11	15.28
Pt/30CoNi-NC-600	22.24	14.72	50.37	12.67
Pt/30CoNi-NC-700	22.75	15.86	49.61	11.78
Pt/30CoNi-NC-800	19.35	16.04	50.77	13.84

Table S4. Summary of fitting results for Pt 4f XPS spectra of different catalysts.

Samples	Pt^0			Pt^{2+}		
	BE (eV)		Relative intensity (%)	BE (eV)		Percentage content (%)
	Pt $4f_{7/2}$	Pt $4f_{5/2}$		Pt $4f_{7/2}$	Pt $4f_{5/2}$	
Pt/30CoNi-NC	71.58	74.88	68.00	72.79	76.09	32.00
Pt/30CoNi-NC-600	71.57	74.87	70.67	72.79	76.09	29.33
Pt/30CoNi-NC-700	71.61	74.91	72.12	72.88	76.18	27.88
Pt/30CoNi-NC-800	71.67	74.97	72.94	72.97	76.27	27.06

Table S5. ECSA of the samples before and after heat treatment.

Samples	ECSA ($m^2\ g^{-1}$)
Pt/30CoNi-NC	41.81
Pt/30CoNi-NC-600	22.48
Pt/30CoNi-NC-700	25.4
Pt/30CoNi-NC-800	12.99
Pt/30CoNi-NC-900	7.86
